# Capacity Strengthening Undertaking—Farm Organized Response of Workers against Risk for Diabetes: (C.S.U.—F.O.R.W.A.R.D. with Cal Poly)—A Concept Approach to Tackling Diabetes in Vulnerable and Underserved Farmworkers in California

**DOI:** 10.3390/s22218299

**Published:** 2022-10-29

**Authors:** Angelos K. Sikalidis, Aleksandra S. Kristo, Scott K. Reaves, Franz J. Kurfess, Ann M. DeLay, Kathryn Vasilaky, Lorraine Donegan

**Affiliations:** 1 Nutrition Program, Department of Food Science and Nutrition, California Polytechnic State University, San Luis Obispo, CA 93407, USA; 2 Cal Poly Personalized Nutrition Research Group, California Polytechnic State University, San Luis Obispo, CA 93407, USA; 3 Center for Health Research, California Polytechnic State University, San Luis Obispo, CA 94307, USA; 4 Department of Computer Science and Software Engineering, California Polytechnic State University, San Luis Obispo, CA 93407, USA; 5 Department of Agriculture Education and Communication, California Polytechnic State University, San Luis Obispo, CA 93407, USA; 6 Department of Economics, Orfalea College of Business, California Polytechnic State University, San Luis Obispo, CA 03497, USA; 7 Department of Graphic Communication, California Polytechnic State University, San Luis Obispo, CA 93407, USA

**Keywords:** agriculture, artificial intelligence (AI), machine learning, farm workers, type 2 diabetes mellitus (T2DM), nutrition, practical decision making

## Abstract

In our project herein, we use the case of farmworkers, an underserved and understudied population at high risk for Type-2 Diabetes Mellitus (T2DM), as a paradigm of an integrated action-oriented research, education and extension approach involving the development of long-term equitable strategies providing empowerment and tailored-made solutions that support practical decision-making aiming to reduce risk of T2DM and ensuing cardiovascular disease (CVD). A Technology-based Empowerment Didactic module (TEDm) and an Informed Decision-Making enhancer (IDMe) coupled in a smart application (app) for farmworkers aiming to teach, set goals, monitor, and support in terms of nutrition, hydration, physical activity, sleep, and circadian rhythm towards lowering T2DM risk, is to be developed and implemented considering the particular characteristics of the population and setting. In parallel, anthropometric, biochemical, and clinical assessments will be utilized to monitor risk parameters for T2DM and compliance to dietary and wellness plans. The app incorporating anthropometric/clinical/biochemical parameters, dietary/lifestyle behavior, and extent of goal achievement can be continuously refined and improved through machine learning and re-programming. The app can function as a programmable tool constantly learning, adapting, and tailoring its services to user needs helping optimization of practical informed decision-making towards mitigating disease symptoms and associated risk factors. This work can benefit apart from the direct beneficiaries being farmworkers, the stakeholders who will be gaining a healthier, more vibrant workforce, and in turn the local communities.

## 1. Background/Introduction

The pursuit of health and well-being extends life quality and expectancy for individuals while fostering positive societal and financial externalities for communities and nations at large. Achieving good health outcomes is challenging in the face of multiple constraints including finances, time, access to services and information [1,2]. While increasing healthcare costs are affecting disproportionally certain individuals and communities, healthcare systems are often supplemented with scattered community programs, targeting particularly disadvantaged communities. However, health statistics on quality of life, well-being, chronic disease burden in the US and globally indicate that desired health outcomes remain elusive [1]. Without viable and long-term healthcare solutions, individuals are left with little recourse, thus in need for alternative approaches to prevent and manage disease, and/or achieve optimal health.

It is important to consider a different approach aiming to enhance healthcare, whereby personalized, adaptive, and integrated smart solutions, paired with real-time/real-life monitoring, aid individuals in making informed practical health decisions and preventing and/or managing chronic disease. This novel approach employs artificial intelligence (AI) and other machine learning techniques (including supervised and reinforcement learning) to help predict outcomes and the drivers of these outcomes, while interfacing with a custodial cloud of experts for fine-tuning and feedback. Such health strategies and assets may well be the way of the future as they empower individuals, account for their diversity of needs, challenges, and opportunities, and grant them ownership of their health.

In the project presented herein, we present the concept of developing a Technology-based Empowerment Didactic module (TEDm) and an Informed Decision-Making enhancer (IDMe) coupled in a smart application (app) for farm workers in California at disproportional risk for Type 2 Diabetes Mellitus (T2DM) with limited access to consultation and healthcare. Additionally, refining and validating the IDMe based on anthropometric/clinical/biochemical parameter testing and monitoring of dietary/lifestyle behavior and nutrition/health-goal achievement degree can supplement and optimize the work. Farm workers constitute a population at significantly higher risk for T2DM [3,4,5] due to genetics, race, and lifestyle elements including improper type of physical activity causing physical stress, irregular sleeping patterns/disrupted circadian rhythm, irregular meal timings, irregular distribution of calories over meals, and an overall suboptimal diet quality and quantity [6,7,8]. The TEDm-IDMe app, would incorporate nutrition education, diabetes education and life-style changes recommendations, deliver those to the participants and set goals based on clinical nutrition and T2DM risk assessment via anthropometric/body composition measurements, extensive blood biochemistry assessing metabolic and inflammatory status, i.e.,: cholesterol (HDL-c, LDL-c and total), atherogenicity index, total triglycerides (TAG), Fasting Plasma Glucose (FPG), Glycosylated Hemoglobin (HbA1c) and C-reactive protein (CRP) and clinical data including heart rate, blood pressure, health status, medications, tobacco and alcohol use. Next, individualized goals are determined, and extent of achievement can be monitored. TEDm-IDMe app would use machine learning (AI/ pattern recognition and comparison to recommendations and cut-offs) and back/re-programing to dynamically provide regular feedback to participants in assisting with achieving their goals, while self-improving. The biomarkers measured are also appropriate for cardiovascular disease (CVD) risk. Development and modes of information delivery for TEDm considers the distinctive idiosyncratic characteristics (opportunities and limitations) of the farmworkers’ setting and lifestyle.

The scope of our work is to enhance optimal practical decision making pertinent to health issues involving lifestyle decisions, for populations with limited access to knowledge and health support. Our approach presented here, is aiming at empowering traditionally and typically underserved population in disadvantaged positions towards chronic disease development in part due to lifestyle and work conditions, significantly relevant to diet and nutrition. Along those lines this approach is innovative in terms of establishing an interdisciplinary tactic to address the issues and lift barriers towards a more equitable delivery of health support, in creating a healthier and more vibrant workforce.

### 1.1. Cost of Type 2 Diabetes Mellitus in the US and CA

According to data from the American Diabetes Association, in the US, in 2015, 30.3 million Americans (9.4% of the census population) had diabetes, with 95% of the cases estimated to be T2DM. An estimated 7.2 million diabetes patients were undiagnosed [3,4,5,6]. Total costs of diagnosed diabetes in the US in 2017 was $327 billion, of which $237 billion for direct medical costs and $90 billion due to reduced productivity [6]. After adjusting for population, age and sex differences, average medical expenditures among people with diagnosed diabetes were 2.3 times higher than projected expenditures in the absence of diabetes [6,7]. In California, approximately 9% of adults have been diagnosed with diabetes, while a staggering 46% including about 1/3 of those under 40 years old are prediabetic, with consistently elevated blood glucose levels, that will most likely develop into frank T2DM. Combined, this amounts to 55% of the State’s adult population being directly affected by diabetes [7]. T2DM incidence spread has increased by 32% in California in the past decade according to State statistics [7]. Treatment of diabetes costs government, private insurers, and patients approximately $37.1 billion per year in California alone, for expenses including doctor visits, testing, medication, surgery, and hospitalization costs. Overall diabetes-generated costs are projected to increase in the coming years both in California and the entire US [7].

### 1.2. Type 2 Diabetes Mellitus among Agriculture Workers

The total prevalence of type 2 diabetes among US agricultural workers is unknown, but it is likely to be similar or higher to that found within US Hispanic population with similar acculturation levels. A study using administrative data collected from 164 Migrant Health Centers on more than 793,000 agricultural worker patients in 2012, reported a prevalence rate of 7.8% for type 1 and type 2 diabetes combined among patients of all ages. These data may well underestimate however the true prevalence of diabetes, based on Health Center billing practices.

Stress is an important contributor to the development of T2DM via physiological effects of stress on blood glucose modulation. Agricultural workers typically exhibit high stress levels due to food insecurity, work type and migration challenges. Fasting blood glucose has been associated with perceived stress among migrant agricultural workers [8] while job strain is shown to be an independent risk factor for T2DM among working men and women, regardless of lifestyle choices [9]. Irregular sleep patterns also tend to aggravate blood glucose control and increase insulin resistance. A systematic review of 59 indigenous populations around the world found that indigenous groups in North America had the highest prevalence of type 2 diabetes, with increasing acculturation contributing to higher rates of diabetes [10]. The risk for diabetes increases in impoverished, “obesogenic” environments, where unhealthy food is inexpensive and accessible and meal patterns are irregular.

### 1.3. Feasibility of Education and Technology Use for Disease Risk Attenuation

Research with a male migratory population of agricultural workers in South Carolina indicated that 81% of the 80 participants had a smartphone and that most participants were very positive of the idea to use mobile technologies for management of hypertension and/or diabetes [11]. Workers unfamiliar with mobile devices also indicated a willingness to participate in mHealth (mobile Health) programs if they had a tutorial. A quasi-experimental study with migrant agricultural workers in Virginia found that community health workers (in Spanish: promotores de salud), could screen for diabetes in this population as effectively as registered nurses [12]. Thus, farmworker screening programs could potentially increase reaching more workers.

## 2. Methodology and Contextual Approach

### 2.1. The Technology-Based Empowerment Didactic Module (TEDm)

Adults approach and engage in learning differently from young learners [13]. The literature suggests adult learners have six significant tenets to learning: (1) they need to know why they must learn something, (2) they need to be viewed as capable, (3) they come with a lot of experience, (4) they are ready to learn, (5) they are focused on the real-world, and (6) they are primarily intrinsically motivated [14]. Familiarity with these six keys can lead to more success in planning and facilitation of adult training programs. Ota et al., recommend adult education be focused on experiential learning methods for maximal effect [13]. Specific methodologies proven successful with adult learners include: lecture, discussion, experiential learning, problem-based learning, storytelling, networking, role-play, educational games, and case studies [13,14,15,16,17].

Educational programming for farm labor presents the need for additional layers of planning. Roka et al., prepared a training model for farm labor supervisors in Florida and proposed the need to develop partnerships with stakeholders, conduct a needs assessment, create, and disseminate curriculum in both English and Spanish, and regularly revise the program based on evaluations [17]. The four workshop themes addressed in the study were packaged into a two full day format while also into a four half-day format. The trainees’ preference was to adhere to a two-day schedule, likely to maximize time away from work.

Madden explained the importance of Hispanic culture to the workplace, but the elements must be considered with training program development. First, Hispanic culture is very paternalistic and hierarchical [18]. The foreman is the head of the work family and workers give them their allegiance, often looking to them as mentors. Training programs must incorporate foremen into the delivery model to secure buy-in. Secondly, many in farm labor have little formal education and may struggle with basic academic skills. Thus, materials must be tailored to the audience, be written in both English and Spanish, free of complex language, and rich in visuals to enhance and convey meaning. Videos can also be a great tool to share information, while delivery of information using printed materials and videos sent in a personal device is less likely to interfere with work schedule. Finally, Hispanic workers take great pride in their work and appreciate seeing how their work touches others, including other workers and their own families. With carefully designed and culturally appropriate training programs, the importance of connection with others in the context of participating in an educational intervention that empowers and offers opportunities for health improvement can help motivate the participants to show compliance with the program and to want to sustain the ensuing benefits as a means of contributing to healthier families and more vibrant communities through their personal improvement and development.

Taking these points into account, the development of educational materials that consider the idiosyncrasies, needs and potential of this population is important. Utilizing a variety of methods in the delivery of the educational material as appropriate is also key, while employing novel technology in doing so, and keeping the materials and information delivery accessible utilizing the mobile technology is the premise upon which the TEDm is founded.

In this context, scripted instructional training modules complete with key learning objectives, content, teaching and learning methods, opportunities for application, and assessment should be developed. Instructional training modules will feature content important feature topics related to: personal nutrition, making healthy food choices, dealing with cravings, exercise programs, sleep, financial outcomes associated with personal health investment (Return On Investment). Modules will be infused with opportunities for personal reflection, goal setting based on personal health data, as well as opportunities for discussion with small groups where Student Health Ambassadors and foremen will also be included. Modules will be delivered by trained Student Health Ambassadors in live training sessions at field locations. Sessions feature a variety of andragogy teaching methods including lecture, discussion, experiential learning, problem-based learning, storytelling, networking, role play, educational games, and case studies. Stakeholders are involved in planning contributing observations/anecdotal evidence regarding the specifics of site, population limitation and opportunities as well as in implementation by providing population access and evaluation by discussing with us our findings and any correlated observations regarding improvements in productivity and workforce retention. The developed app incorporating these materials while creating an interface, becomes available to participants through a smart-phone platform to assist with helping participants develop healthy habits.

### 2.2. The Informed Decision-Making Enhancer (IDMe)

Mobile devices, in particular smartphones and/or wearables, constitute an essential tool for the target population, and used both for personal communication as well as coordination of work-related activities. Health information technology holds significant potential for engaging individuals in managing their health providing tools to track, manage, and interpret personal heath metrics. These tools can empower participants to ask questions, communicate concerns, identify, and assess alternatives, reflect on progress, and alter health behavior. Currently the reach of consumer health informatics technologies among underserved groups including racial/ethnic minorities and low-income individuals remains especially problematic [19]. Participants in our study will be provided with appropriate wearables for the deployment of both the TEDm and IDMe as well as physical activity monitors. Typically, our target group possesses smartphones and are tech-savvy as per the use of associated technologies, while inclined to assign a high value to new trends in the acculturation process, thus willing to acquire new technical knowledge. Relevant tutorials will be provided as needed to facilitate compliance and enhance the participant experience.

Literature review indicates that mobile apps and related technology can positively affect health aspects, in particular weight loss, as well as diabetes prevention and treatment [20,21,22,23,24,25,26,27,28,29,30]. Similarly, an initial literature review indicates a significant number of publications on the use of Artificial Intelligence and Machine Learning techniques for diabetes prediction, prevention, and treatment [28,29,31,32,33,34,35,36,37,38,39,40,41,42,43,44,45,46,47].

### 2.3. App Development and Infrastructure

For the app development, we use a variation of the commonly used “agile development” method. In this method, a small team of software developers, usability evaluators and domain experts work on successive versions of the app. In short periods (“sprints”) of 2–6 weeks, improvements to the app are made and then tested through usability evaluations by actual users. Feedback enters the next development cycle, until the feature set of the app is considered sufficient for a release to the broader user community. Even after this release, improvements continue to be made based on user feedback, changes in technology, performance aspects, monitoring health data, updated clinical/biomarker data. Clinical/biomarker/metabolomics assessment constitutes de facto results-based monitoring and evaluation of the entire extension/education process. As the TEDm-IDMe is implemented, comprehensive health assessment will reflect the progress of behavior-change in reference to chronic disease risk and support finetuning of the TEDm-IDMe.

Through interviews and surveys, we collect information on the background of the target population with respect to their use of related apps (e.g., nutrition tracking, health care), their awareness regarding nutrition and health risks, their openness towards using mobile apps in this context, and their expectations, preferences, and dislikes of potential features of such an app. The expected outcome of this step is a document detailing the features of the proposed app, the translation of the features into requirements that guide the software development, and the identification of evaluation criteria to determine if the requirements have been met, and how the app addresses the needs of the users as expressed in the features.

*App Design and Development:* Based on the requirements identified in the previous step, developers work on the following tasks:

1. Create artefacts such as design sketches, wireframes, and initial mockups that demonstrate the way the features of the app are presented to the users. As the work proceeds, these initial prototypes are expanded with respect to the addition of features, the revision of the appearance, and the performance from a user’s perspective (e.g., the response time for actions initiated by the user).
2. Write code to implement the functionality of the app. In addition to the front end (the part of the app that is visible to the user), there are components performing computational activities behind the scenes. This includes queries to nutritional data bases, collection and storage of user behavior information, generation of recommendations regarding nutrition and behavior modification, and administrative functions such as account management, privacy, and security aspects.
3. Develop the infrastructure for the app. The app is to utilize cloud computing and Web services to connect to nutrition data bases, knowledge repositories, data analysis and machine learning tools, and related components. In addition, the collection and management of user data rely on Web infrastructure. A frequently used tool for such purposes is Google’s Firebase mobile development platform specifically intended for fast, efficient, and secure mobile development.

*Usability Evaluation:* As indicated above, user feedback is considered throughout the development process. Widely used practices for software engineering and mobile development are followed [48,49,50,51,52].

1. Features: Desirable aspects of the app, formulated in a language suitable for the intended user population.
2. Requirements: More formal specification of the features and functionality of the app, formulated for the use of the software developers.
3. Evaluation Criteria: Ideally, these are objective and measurable characteristics of the implemented prototype or system. Within the limits of privacy and technology constraints, we use metrics including time spent with the app, queries made about nutrition, data entry activities. Especially for user interfaces, in-practice user feedback in the form of scales (expressing user satisfaction and similar criteria) or text is commonly used.

On a regular basis, the respective version of the app against the requirements, using the evaluation criteria defined is to be assessed and evaluated. This addresses several aspects:

1. Usability and user experience: Does the current version of the app provide the expected features at that stage? How well can users utilize those features? Are the users satisfied with the way they interact with the app? What problems do users encounter, and what suggestions for improvement do they have.
2. Core functionality of the app: Does the app deliver the expected results? Are these results correct and complete (no missing information)?
3. Infrastructure: Does the app communicate/interact with respective infrastructure as specified?

In the initial stages of development, the emphasis is mainly on the interaction with the main features of the app, and less with performance aspects like response time. In later stages, more emphasis is given to detailed graphic design issues, performance, and stability.

Deployment: For the early versions of the app (sketches, mockups, and partially functional prototypes), development devices are utilized to conduct usability evaluations and collect feedback.

Validation: Ongoing validations of aspects such as usability, functionality, and performance of the app together with the respective infrastructure are to be conducted as well as analysis of the overall outcome for the approach in terms of efficacy through objective clinical assessment.

Clinical assessment constitutes de facto results-based monitoring and evaluation of the entire extension/education process. As the TEDm-IDMe is implemented clinical assessment over time-points demonstrates objectively, in a quantifiable manner, the progress in terms of behavior change as it relates to specific risk parameters for T2DM and ensuing CVD. Typical clinical health indices describing risk for cardiovascular disease and diabetes will be assessed including energy expenditure, body composition, blood pressure, fasting plasma glucose, blood lipid panel, and biomarkers validating dietary intakes per food groups such as red blood cell fatty acids, serum alkylresorcinols, carotenoids and L-carnitine.

### 2.4. Assumptions

1. We assume that farmworkers as indicated by the literature but also common experience, field perception and anecdotal evidence and stakeholder input will have low level of nutrition knowledge and T2DM/CVD knowledge.
2. Research has demonstrated that farmworkers would be positive towards using mobile devices to improve their health as it relates to nutrition and T2DM/Hypertension/CVD.
3. Our population is typically familiar with smart devices but even the few who are not, are still positively predisposed to learning and are found in a conducive environment which could teach them through their interaction with peers.

### 2.5. Hypothesis

We hypothesize that improving knowledge/attitude towards nutrition and other lifestyle parameters affecting T2DM/CVD risk of farmworkers in combination with monitoring and personalized/tailored feedback and customizable help with practical decision making towards health, could result in lower risk for T2DM/CVD, higher productivity and labor retention and an overall more productive and effective labor force.

**Figure 1:**
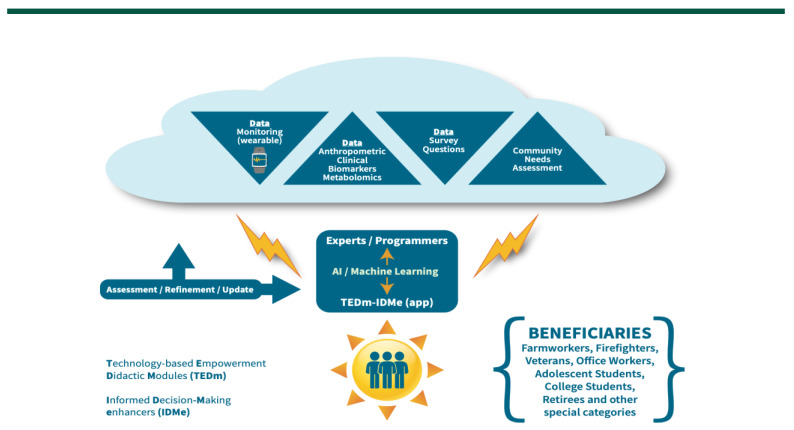
Conceptual graphical schematic of the TEDm-IDMe app approach.

An overall graphical schematic approach on the concepts discussed is provided below with Figure 1. The approach while it uses the paradigm of farmworkers focusing on this population in particular, it can be also followed and applied to other beneficiaries who may be experiencing particular challenges and are thus in a disadvantaged and underserved status due to idiosyncratic lifestyles and work environments/demands.

## 3. Perspectives and Conclusions

Our goal is to lay the foundation for capacity building and the establishment of an approach that will produce comprehensive methods and services through applied research for personalized health monitoring and practical decision making. Thus, this work is significant since the approach followed promotes innovation and interdisciplinarity as it induces ingenuity and creative thinking towards integrating intellectual capital, various specialties and disciplines as well as combines methods and procedures to generate TEDm-IDMe app bundle(s). These applications function as programmable tools that can continuously learn, adapt, and tailor their educational and counseling services to users’ needs. The applications also consider users’ limitations/opportunities, health, and behavior evaluation to help with optimization of practically informed decision making aimed at minimizing risk of chronic disease (such as T2DM and ensuing cardiovascular disease). Addressing the complexity and idiosyncrasies of various target groups is a method of transferring power and responsibility in terms of informed health behavior to the individual in a personalized and continuously evolving way. Systems that include a variety of methods in terms of information acquisition and promote personalized/precision nutrition are generally considered the way of the future both for dealing most effectively with disease but also optimizing health and wellbeing [53,54,55,56,57]. Traditional food and dietary practices and use of technological devices have been seen to improve health outcomes [58,59,60] and our approach may combine the two in the population selected. Limitations of the approach include the extensiveness of information needed and the integration challenges for generating health recommendations. Specific strengths include the comprehensive approach and the personalized precision nutrition and lifestyle considerations.

Moreover, specifically a high level of interdisciplinarity is achieved in this approach by creating possibilities for a hub of experts from fields including nutritional biochemistry/metabolism, metabolomics, communication and education, computer science and software engineering, business, and graphic design. Our aim is to address critical aspects of health assessment, monitoring and data validation with the aid of advanced technology that will be tailor-made and constantly evolving to mirror the needs of its users.

## Data Availability

Data/outcomes available at: nutritioncenter.link.
